# Genetic insights and emerging therapeutics in diabetic retinopathy: from molecular pathways to personalized medicine

**DOI:** 10.3389/fgene.2024.1416924

**Published:** 2024-08-22

**Authors:** Xiaoting Pei, Duliurui Huang, Zhijie Li

**Affiliations:** Henan Eye Institute, Henan Eye Hospital, Henan Provincial People’s Hospital, People’s Hospital of Henan University, People’s Hospital of Zhengzhou University, Zhengzhou, China

**Keywords:** diabetic retinopathy, genetic susceptibility, personalized treatment, genetic variations, gene therapy

## Abstract

Diabetic retinopathy (DR) is a major complication of diabetes worldwide, significantly causing vision loss and blindness in working-age adults, and imposing a substantial socioeconomic burden globally. This review examines the crucial role of genetic factors in the development of DR and highlights the shift toward personalized treatment approaches. Advances in genetic research have identified specific genes and variations involved in angiogenesis, inflammation, and oxidative stress that increase DR susceptibility. Understanding these genetic markers enables early identification of at-risk individuals and the creation of personalized treatment plans. Incorporating these genetic insights, healthcare providers can develop early intervention strategies and tailored treatment plans to improve patient outcomes and minimize side effects. This review emphasizes the transformative potential of integrating genetic information into clinical practice, marking a paradigm shift in DR management and advancing toward a more personalized and effective healthcare model.

## Highlights

• Highlighted the critical role of genetic susceptibility in the pathogenesis of diabetic retinopathy.
• Identified specific genes and genetic variations associated with increased disease risk, offering new perspectives for early intervention.
• Explored personalized treatment strategies based on genetic information, providing innovative methods to improve patient outcomes.
• Emphasized the key role of interdisciplinary collaboration in transforming genetic insights into effective treatment plans.

## 1 Introduction

Diabetic retinopathy (DR) is a severe complication affecting the vision of millions of diabetic patients worldwide. As the most common ocular complication of diabetes, the incidence of DR is increasing with the rising diabetic population, posing a significant public health burden. Without timely diagnosis and treatment, DR can progress to diabetic macular edema (DME) and proliferative diabetic retinopathy (PDR), ultimately leading to significant vision loss or even complete blindness (Pei et al., 2022; Cheung et al., 2022). The impact of DR extends beyond vision impairment, significantly diminishing the quality of life for patients and increasing socio-economic burdens. Thus, understanding its pathogenesis, early diagnosis, and effective treatment are crucial (López et al., 2017).

The occurrence of DR is influenced not only by environmental factors and levels of metabolic control but also by genetic susceptibility. Epidemiological studies and familial analyses have revealed the familial aggregation of DR, suggesting the role of genetic factors in its pathogenesis. Various aspects of the genetics of DR have been explored. Bhatwadekar and colleagues have provided valuable insights into the genetic foundation of the disease through candidate gene studies, association studies, and genome-wide association studies (GWAS), highlighting the genetic contribution to DR (Bhatwadekar et al., 2021).

Genetics holds an irreplaceable position in the study of DR. With the rapid advancements in molecular biology and genetic technologies in recent years, researchers have identified multiple genetic markers associated with the onset and progression of DR (Wang et al., 2020; Hampton et al., 2015; Mishra et al., 2016). The discovery of these genetic factors not only enhances our understanding of the pathophysiology of DR (Sharma et al., 2019) but also provides new tools for early diagnosis and risk assessment of the disease (Isildak et al., 2020; Han et al., 2019).

Despite these advancements, there remains a significant gap in the literature regarding the application of genetic findings in clinical practice. Specifically, how these genetic markers can be effectively integrated into diagnostic and therapeutic strategies for DR has yet to be fully addressed. This research aims to fill this gap by exploring the potential applications of genetic markers in the prediction, diagnosis, and treatment of DR.

**FIGURE 1:**
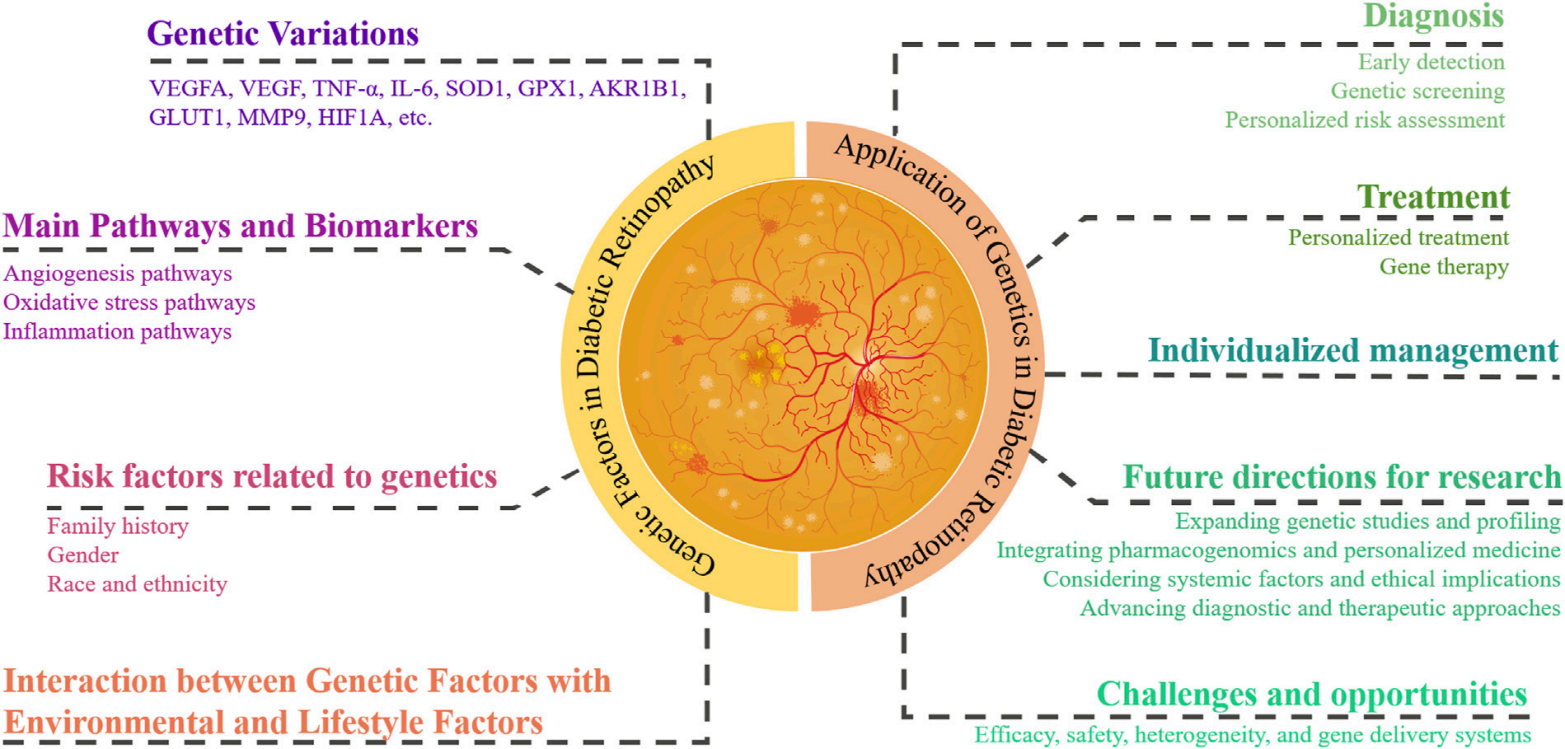
Genetic factors and their application in diabetic retinopathy management. This figure illustrates the integration of genetic information into the understanding and management of DR. Key genetic variations and pathways are highlighted. It outlines genetic risk factors, including family history, gender, and race/ethnicity, and the interaction between genetic and environmental/lifestyle factors. Applications in clinical practice are shown, focusing on diagnosis, treatment, and individualized management. Future research directions and challenges, such as genetic screening, personalized treatment strategies, and addressing ethical, legal, and social issues, are also depicted.

In this review article, we first review the current understanding of the genetic basis of DR, including key findings from GWAS and candidate gene studies. Next, we discuss the potential applications of these genetic discoveries in clinical practice, focusing on early diagnosis and personalized treatment strategies. Finally, we explore the future prospects and challenges in integrating genetic information into the management of DR (Figure 1).

By delving into the genetic foundations of DR, researchers hope to fundamentally change treatment strategies and improve outcomes for patients affected by this vision-threatening complication. This article aims to review the progress in research on genetic susceptibility to DR and discuss its potential applications in prediction, diagnosis, and treatment.

## 2 Methods

This comprehensive literature review was conducted in accordance with the PRISMA Extension for Scoping Reviews (PRISMA-ScR) guidelines. We performed an exhaustive search of several databases, including PubMed, Medline, Web of Science, and the Cochrane Library, covering publications from January 2010 to December 2023. We used a combination of keywords in the title and abstract with the following Medical Subject Headings (MeSH) terms: (“diabetic retinopathy” OR “retina” OR “diabetic macular edema”) AND (“genetics” OR “genetic” OR “epigenetics” OR “gene” OR “hereditary”). The exclusion criteria included (Pei et al., 2022): studies involving animals (Cheung et al., 2022); non-English articles (López et al., 2017); duplicate publications (Bhatwadekar et al., 2021); conference abstracts, commentaries, or reports.

To determine the appropriate time frame for our literature review, we conducted a preliminary search and identified several comprehensive review articles that summarized findings up to the year 2010. To supplement these earlier discoveries, we meticulously reviewed newly published literature from the past decade. Therefore, covering literature from 2010 to 2023 provides a comprehensive overview of the application of genetics in diabetic retinopathy.

To ensure the inclusiveness of our literature collection, we also manually reviewed the references in the original research papers and review articles identified to discover additional studies that were not retrieved through database searches. The literature was evaluated using a double-blind peer review process to guarantee the quality of the selected publications and the reliability of the data.

## 3 Genetic factors in diabetic retinopathy

Genetic susceptibility influences not only the risk of DR but also the pace and severity of its progression. Certain gene mutations can accelerate the disease course, causing rapid development or progression to a more severe state in a shorter timeframe (Imamura et al., 2021; Goonesekera et al., 2015). Understanding the genetic susceptibility to DR is crucial for early detection and targeted treatment.

### 3.1 Genetic variations affecting the risk of diabetic retinopathy

Vascular endothelial growth factor A (VEGFA) is a pivotal gene in promoting angiogenesis, the formation of new blood vessels. Polymorphisms in VEGFA, such as rs699947 and rs2010963, are associated with a higher risk of DR, highlighting genetic predisposition to the disease. These variations affect the gene’s regulatory regions, leading to differential expression levels that exacerbate retinal neovascularization (McDonald and Schatz, 1985; Penn et al., 2008). The VEGF gene encodes a protein crucial for cell growth, proliferation, and differentiation. Genetic polymorphisms affecting VEGF expression and activity also influence its role in DR. Variants in the VEGF gene may alter its binding affinity to receptors or its expression levels, impacting endothelial cell proliferation and angiogenesis in DR (Cheung et al., 2010; Simó et al., 2014; Awata et al., 2002).

Tumor necrosis factor-alpha (TNF-α) is a potent pro-inflammatory cytokine crucial to DR pathogenesis. Genetic polymorphisms in the TNF-α gene are associated with an increased risk of DR. For example, the TNF-α −308G/A polymorphism (rs1800629) is linked to higher TNF-α production and increased DR susceptibility. This polymorphism affects the TNF-α gene’s promoter region, enhancing transcriptional activity and elevating cytokine levels (Sikka et al., 2014). Interleukin-6 (IL-6) is associated with increased vascular permeability and retinal inflammation. Polymorphisms in the IL-6 gene, like the −174G/C polymorphism (rs1800795), are linked to variations in IL-6 expression and DR susceptibility. This polymorphism influences the IL-6 gene’s promoter activity, affecting cytokine production levels (Sun and Guo, 2020).

Superoxide dismutase 1 (SOD1) is crucial for defense against oxidative stress. Variations in the SOD1 gene, such as single nucleotide polymorphisms (SNPs), affect the enzyme’s activity and efficiency. For example, the rs2070424 polymorphism in SOD1 is associated with altered enzyme function and increased susceptibility to oxidative stress-related retinal damage (Saremi et al., 2021). Glutathione peroxidase 1 (GPX1) is a key antioxidant enzyme. Polymorphisms in the GPX1 gene influence enzyme activity and oxidative stress levels in retinal cells. For instance, the rs1050450 polymorphism in the GPX1 gene is linked to reduced enzyme activity and increased oxidative stress, exacerbating retinal damage in DR (Asadi et al., 2024).

Furthermore, GWAS have identified new genetic loci and genes such as AKR1B1, GLUT1, COL18A1, MMP9, HIF1A, and CFH, which are linked to DR susceptibility, though further replication studies are needed to confirm these associations (Imamura et al., 2021; Kaur and Vanita, 2016; Gutierrez et al., 1998; Kowluru et al., 2016; Mohamed et al., 2022; Wang et al., 2013). Research has also explored the relationship between specific gene mutations (e.g., eNOS and α2β1 integrin genes) and DR occurrence in T2DM patients, highlighting the crucial role of these genes in regulating retinal vasculature (Azmy et al., 2012). These discoveries not only enhance our understanding of the genetic basis of DR but also highlight new therapeutic targets and strategies, underlining the transformative potential of personalized medicine in improving the care and management of DR patients.

### 3.2 Main pathways and biomarkers involved in diabetic retinopathy

**FIGURE 2:**
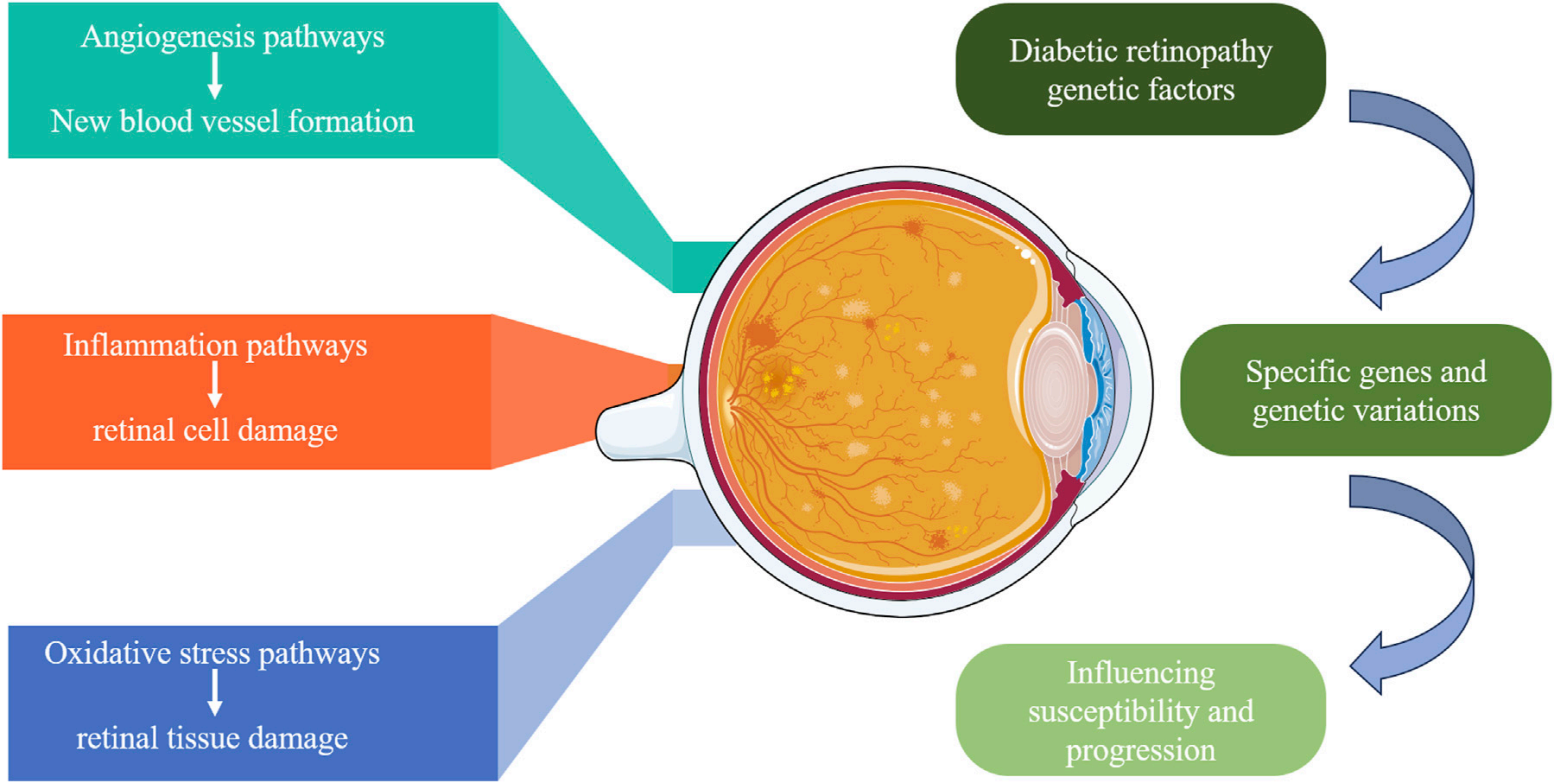
Genetic factors and pathways in diabetic retinopathy. This diagram illustrates the genetic factors contributing to DR. It highlights the specific genes and genetic variations that influence susceptibility and progression of DR, focusing on three main pathways: angiogenesis (new blood vessel formation), inflammation (retinal cell damage), and oxidative stress (retinal tissue damage). These pathways collectively contribute to the development and severity of DR.

DR is a multifactorial disease influenced by various biomarkers and pathways, among which angiogenesis, inflammatory responses, and oxidative stress are key factors in the occurrence and progression of DR (Capitão and Soares, 2016). Figure 2 displays genetic factors and pathways in DR. The main pathways and biomarkers involved in DR are shown in Table 1 **.**

**TABLE 1:** Main pathways and biomarkers involved in diabetic retinopathy.

Pathways	Biomarkers and genes	Role in diabetic retinopathy
Angiogenesis	VEGFA (rs699947, rs2010963), VEGF, PDGF, FGF, Ang-2	Promotes new blood vessel formation, crucial in PDR development
Inflammation	TNF-α (rs1800629), IL-6 (rs1800795), TLR4, IRAK1, TIRAP	Drives retinal inflammation, vascular leakage, and cell death
Oxidative stress	SOD1, SOD2 (rs4880), GPX1, CAT	Leads to cell and tissue damage through excess free radicals
Glucose metabolism	AKR1B1, GLUT1	Causes osmotic stress and cellular damage in the retina
Cell adhesion and extracellular matrix	COL18A1, MMP9	Maintains blood vessel structure and increases vascular permeability
Hypoxia response	HIF1A, eNOS, ICAM-1	Regulates response to low oxygen levels, promoting neovascularization
Complement system	CFH	Regulates immune responses, with variations linked to DR risk

#### 3.2.1 Angiogenesis

##### 3.2.1.1 Angiogenesis and development of diabetic retinopathy

Angiogenesis is crucial in DR development, particularly in PDR. VEGF is a key pro-angiogenic factor that accelerates DR progression by promoting neovascularization. VEGF expression is regulated by factors such as hypoxia and inflammation, which together form a complex regulatory mechanism (Behl and Kotwani, 2015). Clinically, anti-VEGF therapy effectively slows DR progression and is successful in treating PDR and DME (Simó et al., 2014). However, individual responses to anti-VEGF therapy vary significantly, closely related to genetic background. Recent studies suggest that specific gene variants may influence responses to anti-VEGF treatment, explaining the efficacy differences among individuals (Sajovic et al., 2019).

Besides VEGF, other pro-angiogenic factors like platelet-derived growth factor (PDGF) and fibroblast growth factor (FGF) also play significant roles in DR pathology (Gong et al., 2016). Multi-targeted therapies might provide a more comprehensive treatment, especially for patients unresponsive to single anti-VEGF treatments. Further research into the signaling pathways and genetic regulation of angiogenesis could aid in developing new therapies, improving DR prognosis (Zhang et al., 2018).

##### 3.2.1.2 Genetics related to angiogenesis

Polymorphisms in the VEGF gene significantly impact the angiogenic process in DR. For instance, variations like rs699947 and rs833061 in the VEGF gene are significantly associated with DR risk. These polymorphisms affect retinal neovascularization by regulating VEGF expression levels (Sudhesan et al., 2017). Other angiogenic factors like angiopoietin-2 (Ang-2) and FGF also play important roles in DR angiogenesis. Gene variations in these factors may promote retinal pathology by affecting endothelial cell stability and signaling (Loukovaara et al., 2013).

Genetic variations affect the onset and progression of DR and may influence the efficacy of anti-VEGF therapies. Studies found that certain VEGF gene polymorphisms may affect drug efficacy and side effects by regulating VEGF expression or function (Lu et al., 2013; Di Stefano et al., 2015). Identifying these variations through genetic testing allows clinicians to better predict patient responses to anti-VEGF therapies and adjust treatment plans accordingly.

Angiogenesis involves a complex network of genes and signaling pathways. GWAS and next-generation sequencing (NGS) have identified multiple gene loci and signaling pathways related to angiogenesis. These include HIF-1α, eNOS, and ICAM-1, which regulate hypoxia response and neovascularization in the retina (Sharma et al., 2019; Choi et al., 2016).

Thoroughly studying the relationship between these genetic factors and angiogenesis helps scientists and clinicians better understand DR pathology and develop precise diagnostic and therapeutic strategies. This will enhance early detection and prevention of DR and significantly improve patient prognosis and quality of life through personalized treatment.

#### 3.2.2 Inflammatory response

##### 3.2.2.1 Inflammatory response and development of diabetic retinopathy

Inflammation plays a crucial role in DR development. Elevated levels of inflammatory cells and cytokines, particularly IL-6 and TNF-α, in DR patients indicate active inflammatory pathways. These factors drive DR progression by causing vascular leakage, neovascularization, and retinal cell death, highlighting the need for new DR treatments (Rezzola et al., 2020; Madonna et al., 2016).

##### 3.2.2.2 Genetics related to the inflammatory response

Recent studies show that genetic variations in inflammatory genes, particularly TNF-α and IL-6, may accelerate DR development. Degirmenci et al. found that TLR4, IRAK1, and TIRAP gene variations are not directly associated with T2DM risk but are linked to elevated inflammatory markers, suggesting their role in modulating inflammatory responses in DR (Degirmenci et al., 2019). Specifically, TLR4 gene polymorphisms may enhance inflammatory signaling, promoting retinal inflammation and DR progression. TNF-α is a crucial pro-inflammatory cytokine, and its gene polymorphisms play a significant role in DR development. Moemen et al. confirmed that TNF-α is important at all stages of DR (Moemen et al., 2023). For example, the rs1800629 variant in the TNF-α gene is significantly associated with DR susceptibility, potentially increasing TNF-α expression and exacerbating inflammation, retinal damage, and vascular abnormalities (Abhary et al., 2009).

IL-6 gene polymorphisms are also linked to DR risk and progression. A study showed that changes in inflammatory mediators in early DR stages are closely associated with retinal microvascular changes (Feng et al., 2018). Specifically, the rs1800795 variant in the IL-6 gene may increase IL-6 expression, enhancing inflammatory responses and causing retinal cell damage and vascular leakage (Ołdakowska et al., 2022). IL-6 promotes inflammation and synergizes with other cytokines to drive neovascularization and further retinal damage. Giblin et al. demonstrated that NFAT inhibitors reduce inflammatory responses by lowering IL-1β-induced TNF-α and IL-6 levels (Giblin et al., 2021). These findings underscore the significance of controlling inflammation in developing DR treatments.

#### 3.2.3 Oxidative stress

##### 3.2.3.1 Oxidative stress and development of diabetic retinopathy

Oxidative stress, caused by an excess of free radicals, is crucial in DR development and progression (Kang and Yang, 2020). This condition damages cells and tissues, contributing significantly to DR progression by harming cell membranes, proteins, and DNA. Carpi-Santos et al. identified Müller cells as potential targets for DR therapy due to oxidative stress and inflammation (Carpi-Santos et al., 2022). Calderón et al. detailed oxidative stress’s impact on DR and proposed stage-specific treatment strategies (Calderon et al., 2017). Moreover, genetic variations in SOD1 and SOD2 can modify an individual’s oxidative stress response, influencing DR onset (Hovnik et al., 2009). Thus, antioxidants may help prevent and treat DR. Tu et al. found that melatonin alleviates oxidative stress and inflammation in DR by activating the Sirt1 pathway, highlighting melatonin’s promise in antioxidant and anti-inflammatory therapies and offering new management strategies for DR (Tu et al., 2021).

##### 3.2.3.2 Genetics related to oxidative stress

Kang et al. studied the molecular mechanisms, pathogenic roles, and impacts of oxidative stress in DR, noting that cytopathic outcomes result from excessive reactive oxygen species (ROS) generation and subsequent suppression of antioxidant defenses (Kang and Yang, 2020). The study emphasized the critical roles of antioxidant enzymes like SOD and catalase (CAT) in combating oxidative stress and their potential in DR treatment. Variations in SOD1 and SOD2 genes can affect enzyme function, altering individuals’ responses to oxidative stress (Wu et al., 2014). SOD1 and SOD2 polymorphisms play significant roles in DR, with the rs4880 variation in SOD2 significantly associated with DR risk by reducing SOD2 activity, increasing intracellular ROS levels, and leading to retinal cell damage and disease progression (Cecilia et al., 2019).

Additionally, SOD1 gene variations are linked to DR risk, potentially exacerbating oxidative stress by affecting SOD1 function (Wu et al., 2014). Under hyperglycemic conditions, individuals with SOD1 or SOD2 variations may exhibit higher oxidative stress levels, leading to more severe retinal damage (Li et al., 2017). Research into gene-environment interactions is essential for understanding DR’s complex etiology and developing personalized treatment strategies.

Given the close relationship between oxidative stress and genetic variations in DR development, targeting specific oxidative stress-related genes could effectively prevent or treat DR. Understanding oxidative stress mechanisms in DR is crucial for identifying potential therapeutic targets. Antioxidant strategies play a key role in DR treatment, and ongoing research continues to open new avenues for DR management (Fahmy et al., 2021).

### 3.3 Risk factors for DR related to genetics

#### 3.3.1 The role of family history

Research indicates that family history plays a crucial role in the development and progression of DR, suggesting a substantial genetic component in its pathogenesis (Imamura et al., 2021; Santos et al., 2020). Numerous studies have shown that severe forms of DR, such as PDR, tend to cluster in families. The FinnDiane study found a significant familial risk for proliferative retinopathy, with an odds ratio of 2.76 for siblings of probands, highlighting the importance of genetic factors in DR (Hietala et al., 2008). Similarly, the FIND-Eye study reported heritability estimates for DR severity at 27%, underscoring the familial component in DR (Arar et al., 2008). A study of 1,228 type 2 diabetic patients found significant associations between DR and family history of diabetes (Maghbooli et al., 2014).

Understanding family history’s role in DR is vital for developing clinical management and screening strategies. For instance, individualized screening intervals based on familial risk factors may aid in early detection and intervention. In summary, family history is a significant factor in DR development and progression. Future research should explore the genetic mechanisms underlying DR to enhance prevention and treatment strategies.

#### 3.3.2 Gender

The impact of gender on DR is a crucial topic in the research of diabetes complications. Studies suggest that gender influences the incidence and progression of diabetes, as well as the risk, severity, and treatment response of DR (Cherchi et al., 2020; Nakayama et al., 2021). Epidemiological studies indicate that men are more likely than women to develop DR under poor diabetes management (Enikuomehin et al., 2020; Li et al., 2020). The influence of gender on the severity of DR yields varied findings (Sattar and Tufaili, 2020). Some studies show men have a higher risk of progressing to advanced DR stages, while others find certain subgroups of women are more prone to specific DR forms, such as diabetic macular edema (Li et al., 2020; Liu et al., 2006). Gender may also influence DR treatment effectiveness. Preliminary evidence, though limited, suggests gender differences in responses to treatments like laser photocoagulation or anti-VEGF, highlighting the need for more clinical studies to provide personalized treatment options (Li et al., 2020; Maurya et al., 2021). These differences may relate to sex hormones, genetic background, lifestyle, and their interactions with the environment (Khan et al., 2020).

Future research should explore how gender influences DR risk and treatment responses through biological mechanisms, hormones, genetic factors, and lifestyle (Cassidy et al., 2020). Additionally, studies should assess the impact of gender differences on DR screening, prevention, and clinical management to develop more accurate and effective treatments.

#### 3.3.3 Race and ethnicity

Research shows significant disparities in DR prevalence, progression rate, and severity among racial and ethnic groups, likely influenced by genetic factors, socio-economic status, lifestyle, and diabetes management variations (Olvera-Barrios et al., 2023; Malhotra et al., 2021). Data show that African Americans, Hispanics/Latinos, and certain Asian populations (especially South Asians) are more likely to develop DR than Caucasians (Cheung et al., 2022; Luo et al., 2019). The Multi-Ethnic Study of Atherosclerosis showed that DR prevalence was higher among Blacks (36.7%) and Hispanics (37.4%) compared to Whites (24.8%) and Chinese (25.7%) in the United States (Wong et al., 2006). Studies show that compared to Caucasian diabetic patients, African and Hispanic/Latino patients are more likely to progress to treatment-requiring PDR or DME (Goonesekera et al., 2015; Thomas et al., 2021). Differences in treatment responses among racial and ethnic groups are also important in DR management. Although research is limited, some evidence suggests variability in response to conventional DR treatments, like laser therapy and anti-VEGF medications, among different racial and ethnic groups (Thomas et al., 2021; Kirthi et al., 2021). This variability may require physicians to consider racial and ethnic backgrounds when selecting treatment plans.

In conclusion, race and ethnicity are crucial determinants of DR risk and treatment outcomes. By understanding these disparities, healthcare providers can offer more personalized and effective management strategies for diabetic patients from diverse backgrounds, improving their vision and quality of life.

### 3.4 How genetic variations interact with environmental and lifestyle factors

**FIGURE 3:**
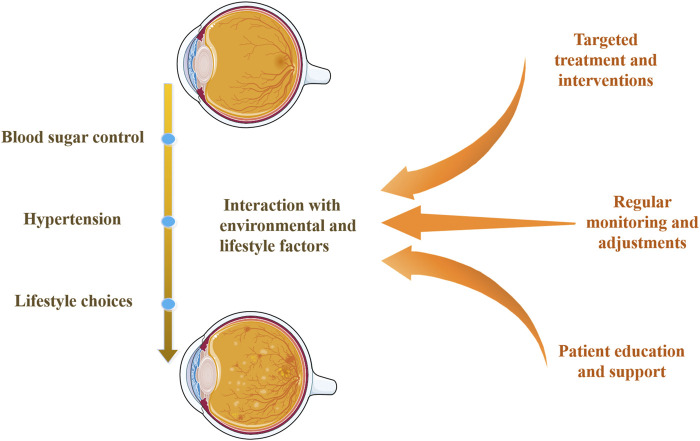
Interaction of genetic variations with environmental and lifestyle factors. This diagram shows how genetic predispositions for DR interact with environmental and lifestyle factors, such as blood sugar control, hypertension, and lifestyle choices. It highlights DR’s multi-factorial nature and emphasizes comprehensive management strategies, including targeted treatments, regular monitoring, and patient education and support.

Exploring the interaction between genetic susceptibility and environmental factors is crucial for understanding genetic predisposition in DR. Figure 3 illustrates the impact of genetic variations interacting with environmental and lifestyle factors on DR. Research indicates that although individuals may carry genetic variations increasing DR risk, a healthy lifestyle and effective blood sugar control can significantly reduce DR development likelihood (Consortium, 2016; Klimentidis et al., 2014; Ye et al., 2021). Blood sugar control is a key environmental factor affecting DR progression, and its interaction with genetic susceptibility can exacerbate retinal damage (Sharma et al., 2019; Ye et al., 2021). Similarly, hypertension can increase DR risk, and certain genetic variations may make individuals more sensitive to blood pressure regulation (Zhao et al., 2022). Additionally, lifestyle choices such as diet, smoking, and physical activity can influence DR risk by regulating metabolic and inflammatory responses (Ye et al., 2021). Therefore, individuals with specific genetic predispositions can reduce DR risk by optimizing blood sugar control, maintaining normal blood pressure, and adopting a healthy lifestyle.

In summary, the interaction among genetic variations, environmental factors, and lifestyle choices is crucial for DR risk assessment and management. Integrating genetic research findings with personalized treatment strategies can significantly optimize DR patient outcomes. Genetic susceptibility profoundly impacts DR, showing great potential for disease prediction, diagnosis, and treatment. With advances in genetic research and precision medicine, more personalized and effective management strategies for DR patients are anticipated, paving new pathways for treating this vision-impairing complication.

## 4 The Application of genetics in the diagnosis and treatment of diabetic retinopathy

Genetics plays a pivotal role in the diagnosis and treatment of DR. Understanding the genetic underpinnings of DR offers valuable insights into its pathogenesis and potential therapeutic targets. The identification of genetic susceptibility provides new avenues for personalized management of DR (Priščáková et al., 2016). Screening for high-risk genetic markers enables early prediction and diagnosis of DR, allowing for the creation of personalized monitoring and treatment plans for patients (Pusparajah et al., 2016; Peiyu et al., 2023). Furthermore, predictions of drug responses based on genetic information help select the most appropriate treatment, avoiding ineffective therapy and reducing adverse reactions, thereby achieving precision medicine.

### 4.1 Application of genetics in diagnosis

The application of genetics in the diagnosis of DR is a rapidly evolving field, poised to revolutionize early detection and risk assessment of this common diabetes complication (Jin et al., 2016). Researchers and clinicians are utilizing insights from genetic studies to identify high-risk populations for DR and implement timely interventions to mitigate the risk of DR progressing to severe vision impairment or blindness.

#### 4.1.1 Genetic markers for early detection

Recent studies have identified several genetic markers associated with an increased risk of DR. Notable genes include VEGFA, involved in angiogenesis, and TNF-α and IL-6, which regulate inflammatory responses. The VEGFA gene plays a crucial role in angiogenesis, with polymorphisms such as rs699947, rs833061, and rs2010963 significantly affecting the extent of retinal neovascularization, a primary driver of PDR. High levels of VEGF not only promote abnormal blood vessel formation but also increase vascular permeability, leading to retinal edema and hemorrhage, further exacerbating vision loss (Bhatwadekar et al., 2021). The TNF-α gene is an important inflammatory regulator, and polymorphisms such as rs1800629 are significantly associated with DR susceptibility. These variations may lead to increased TNF-α expression, triggering and exacerbating retinal inflammation, disrupting the blood-retinal barrier, and causing retinal cell apoptosis and vision impairment (Degirmenci et al., 2019). Similarly, IL-6 gene polymorphisms like rs1800795 affect DR development by regulating systemic and local inflammatory responses. High IL-6 levels not only increase retinal vascular permeability but also induce inflammatory cell infiltration and cytokine release, further damaging the retina (Xiao et al., 2022).

Additionally, polymorphisms in the AKR1B1 gene, which encodes aldose reductase and is involved in the metabolic pathways of diabetic complications, are significantly associated with the risk of retinopathy in diabetic patients. These variations may affect retinal cell responses to oxidative stress, exacerbating retinopathy (Cao et al., 2018). Variations in the ICAM1 and eNOS genes, which play crucial roles in regulating vascular function and inflammatory responses, are also related to the pathogenesis of DR. Research and detection of these gene variations can enhance our understanding of the genetic basis of DR, leading to the development of more precise early diagnostic tools and personalized treatment strategies. With the advancement of genetic testing technologies, these genetic markers will become increasingly widespread in the early detection of DR, potentially significantly improving patient prognosis and quality of life.

#### 4.1.2 Advances in genetic screening

Recent advancements in genetic screening technologies, especially GWAS and NGS, have significantly propelled the identification of genetic factors associated with DR (Sharma et al., 2019). GWAS has identified new genetic loci linked to DR risk, such as STT3B, PALM2, and EHD3, offering new perspectives on the genetic basis of DR (Imamura et al., 2021). Concurrently, NGS technology has enhanced the detection of DR-related genetic variations, uncovering common SNPs, rare gene mutations, and structural variations that may play crucial roles in DR pathogenesis (Jin et al., 2016).

For example, NGS has identified rare mutations in the AKR1B1 and NOS3 genes closely associated with DR progression (Cao et al., 2018). Moreover, integrating GWAS and NGS data allows researchers to map gene networks, revealing interactions and co-regulation among genes, thereby aiding in understanding the complex pathophysiology of DR (Han et al., 2019).

Functional validation experiments, such as gene knockout and overexpression studies, have verified the specific functions of newly discovered genetic variants in DR, providing a scientific basis for new therapeutic strategies (Qin et al., 2017). As genetic screening technologies advance, these newly discovered genetic variations and loci are gradually being applied in clinical practice. Genetic screening of high-risk individuals enables early diagnosis and intervention, reducing disease progression risk. Furthermore, personalized medical plans rely heavily on a comprehensive understanding of patients’ genetic backgrounds.

In the future, combining machine learning and artificial intelligence will enhance the efficiency and accuracy of genetic data analysis, promoting precision medicine. This advancement is expected to significantly improve patient prognosis and quality of life.

#### 4.1.3 Personalized risk assessment

The ultimate goal of applying genetics in DR diagnosis is to achieve personalized medicine. Combining genetic risk factors with traditional ones, like diabetes duration, glycemic control, and blood pressure, enables healthcare providers to develop personalized risk assessment plans for patients. Integrating genetic and clinical risk factors enables comprehensive evaluation of each patient’s DR risk. Based on these assessments, providers can design individualized monitoring and early intervention plans.

For high-risk individuals with VEGFA gene variants, retinal examination frequency can be increased, and early use of anti-VEGF therapy can be considered. Conversely, patients with TNF-α or IL-6 gene variants may benefit from early anti-inflammatory treatments to mitigate inflammation and protect the retina (Agarwal et al., 2016). Personalized risk assessments also guide customized treatment plan development. Selecting drugs and treatment methods targeting specific genetic variations can optimize treatment efficacy and minimize side effects.

Understanding gene-environment interactions is crucial, as factors like diet, exercise, and socioeconomic status can influence DR onset and progression. Studying these interactions enables the formulation of more comprehensive and effective intervention strategies. Advances in genomics, machine learning, and big data analysis will make personalized DR risk assessment more precise and efficient, significantly improving patient prognosis and reducing disease burden (Cabrera et al., 2020a).

### 4.2 Application of genetics in treatment

Incorporating genetics into DR treatment represents a significant advancement in personalized medicine, offering new avenues for developing targeted therapies based on individual genetic profiles (Wang et al., 2020). This approach tailors interventions to genetic determinants of susceptibility, progression, and treatment response in DR, thereby enhancing treatment efficacy and minimizing adverse reactions.

#### 4.2.1 Genetic insights into diabetic retinopathy

Understanding the genetic basis of DR requires identifying specific genes and genetic variations that affect disease risk and severity. Research has identified numerous genes associated with DR, including those involved in angiogenesis (e.g., VEGFA), inflammation (e.g., TNF-α), and oxidative stress (e.g., SOD2), which are key pathways in DR onset and development (Wang et al., 2017; Whitehead et al., 2018).

Abnormal angiogenesis characterizes PDR, and variations in VEGFA, a key angiogenesis regulator, may lead to retinal neovascularization (Abhary et al., 2009; Nakamura et al., 2009), causing retinal hemorrhage and vision loss (Awata et al., 2002). Inflammation is a significant pathological process in DR. TNF-α is a key gene regulating inflammatory responses. Polymorphisms in the TNF-α gene may increase TNF-α expression, triggering chronic retinal inflammation, disrupting the blood-retinal barrier, and exacerbating DR (Sikka et al., 2014).

Oxidative stress plays a crucial role in DR onset and progression. The SOD2 gene encodes an antioxidant enzyme responsible for scavenging intracellular superoxide radicals. Polymorphisms in the SOD2 gene, such as rs4880, are significantly associated with DR risk. Reduced SOD2 function can enhance oxidative stress, damage retinal cells, and promote DR progression (Al-Kateb et al., 2008).

Comprehensive analysis of these gene variations provides a more complete understanding of the genetic basis of DR. Advanced genomic technologies, such as GWAS and NGS, enable researchers to uncover complex gene networks and biological pathways related to DR. These discoveries offer new targets for early diagnosis and prevention of DR and provide a scientific basis for developing personalized treatment plans.

#### 4.2.2 Personalized treatment strategies

The goal of using genetic information in DR treatment is to develop pharmacogenomic strategies that optimize treatment efficacy and reduce toxicity by tailoring therapies to patients’ genetic profiles. Anti-VEGF therapy, a standard treatment for PDR and DME, varies in efficacy and risk of adverse reactions among individuals due to genetic variations in the VEGFA gene (Wu et al., 2017). Identifying patients with genetic predispositions to respond well to anti-VEGF drugs allows clinicians to provide more accurate treatment.

Pharmacogenomics in DR treatment primarily guides drug selection and dosage adjustments through genetic testing. Polymorphisms in the VEGFA gene, such as rs833061 and rs2010963, are linked to the efficacy of anti-VEGF treatments. Studies suggest these genetic variations influence VEGF expression levels and anti-VEGF drug effectiveness, thus affecting treatment outcomes (Wu et al., 2017). Genetic testing allows clinicians to predict which patients will respond better to anti-VEGF therapies and adjust treatment plans accordingly, enhancing effectiveness and safety.

Besides anti-VEGF therapy, anti-inflammatory treatments are crucial for managing DR. Polymorphisms in TNF-α and IL-6 genes are closely related to inflammatory responses, influencing patients’ responses to anti-inflammatory drugs. For example, the rs1800629 polymorphism in the TNF-α gene affects patients’ responses to anti-TNF-α drugs. Genetic testing can identify patients likely to respond well to anti-inflammatory treatments, allowing more precise interventions (Sikka et al., 2014). Similarly, IL-6 gene variations can guide anti-IL-6 drug use to reduce retinal inflammation.

Polymorphisms in the SOD2 gene, such as rs4880, may affect patients’ responses to antioxidant treatments. By detecting these genetic variations, clinicians can select appropriate antioxidants, such as vitamins C and E, to mitigate oxidative stress in the retina and slow the progression of DR (Wang et al., 2010).

Personalized treatment strategies include integrating multiple treatments, not just single-drug therapies. Combining genetic testing results with clinical characteristics allows clinicians to develop comprehensive treatment plans. For example, some patients may require a combination of anti-VEGF, anti-inflammatory, and antioxidant therapies. Integrating these methods can more effectively control DR progression. Additionally, lifestyle interventions, such as dietary adjustments and exercise, can be personalized based on patients’ genetic backgrounds to enhance treatment outcomes.

Advances in genomic technologies will likely identify more DR-related genetic variations, promoting the development of personalized medicine. Continuous research and clinical practice will enhance the precision and effectiveness of personalized treatment strategies, significantly improving DR patient prognosis and quality of life (Hampton et al., 2015).

#### 4.2.3 Gene therapy for diabetic retinopathy

##### 4.2.3.1 Prospects and applications of gene therapy

Gene therapy leads innovative approaches for managing DR, targeting the disease’s genetic and molecular roots to offer solutions beyond symptomatic relief. Recent advancements in gene therapy for DR include strategies such as anti-angiogenic, anti-inflammatory, oxidative stress reduction, and neuroprotection, heralding a new era of personalized treatments for diabetic eye diseases.

Anti-angiogenic gene therapy targets key factors like VEGF, crucial in proliferative DR development. This strategy promises to halt disease progression by directly addressing the neovascularization process (Whitehead et al., 2018; Robles-Rivera et al., 2020). Anti-inflammatory gene therapy modulates inflammatory pathways by targeting specific cytokines and chemokines, addressing a fundamental disease driver (Tang et al., 2023). Gene therapy addressing oxidative stress seeks to upregulate antioxidant enzymes like superoxide dismutase and catalase to mitigate retinal oxidative damage and prevent further deterioration. This strategy is crucial in early DR stages, targeting oxidative stress pathways to preserve retinal health (Kang and Yang, 2020). Neuroprotective gene therapy focuses on preserving retinal ganglion cells and other neural components, addressing DR’s neurodegenerative aspects to potentially prevent vision loss (Rolev et al., 2021). This approach emphasizes the need for early intervention and the potential of gene therapy to alter DR’s course by targeting its underlying causes rather than just its symptoms.

These gene therapy approaches highlight a shift toward personalized and targeted treatments for DR, leveraging genetic research and technology to transform patient care in diabetic eye disease. Advancements in genomic technology will likely identify more genetic markers associated with disease risk and treatment response, offering promising prospects for DR management.

##### 4.2.3.2 Limitations and challenges of gene therapy

Gene therapy is a cutting-edge approach for managing DR by targeting its genetic and molecular roots. However, it faces significant limitations and challenges. Effectively delivering therapeutic genes to target retinal cells remains a major challenge. The retina’s complex, delicate structure makes efficient and precise gene delivery difficult without causing damage (Wang et al., 2020). Commonly used viral vectors can trigger immune responses or inflammation, potentially worsening the condition (Ting and Martin, 2006). The long-term efficacy and stability of gene therapy remain uncertain. While some strategies show initial promise, their duration is unclear, raising concerns about the need for repeat treatments. Repeat treatments could increase adverse effects and complicate patient compliance (Mizukami et al., 2011). Gene therapy carries inherent risks, such as integrating therapeutic genes into non-target sites in the genome, potentially causing unintended consequences like tumorigenesis or other genetic disorders. Immune responses to viral vectors may also limit the feasibility of repeat treatments (Agarwal et al., 2016).

Genetic and epigenetic diversity among DR patients presents a significant obstacle. Personalized gene therapies effective for one individual may not be applicable to another due to genetic variations. This requires extensive genetic screening and tailored treatment plans, which can be time-consuming and expensive (Whitehead et al., 2018). On the regulatory and ethical front, developing and implementing gene therapy for DR faces stringent scrutiny due to high risks. Ethical concerns about gene modification and its long-term effects on future generations pose major challenges that could delay adopting these therapies (Campa et al., 2017). High costs of researching, developing, and treating with gene therapies may limit their accessibility, especially in low- and middle-income countries. Ensuring affordability and broad accessibility of these advanced therapies is crucial. Despite advances in genetic research, our understanding of DR pathogenesis is still evolving. This incomplete knowledge may hinder developing fully effective gene therapies. Continuous research is needed to uncover more about disease mechanisms and identify new therapeutic targets (Hanis and Hallman, 2006).

These limitations highlight the complexity of developing gene therapies for DR. While the potential benefits are significant, overcoming these challenges requires further research, technological advancements, and multidisciplinary approaches to make gene therapy a reliable and accessible treatment for DR patients.

##### 4.2.3.3 Future directions for diabetic retinopathy gene therapy

The future of gene therapy for DR involves overcoming current limitations and expanding our understanding of the disease. Developing efficient and targeted gene delivery systems that minimize immune responses and maximize therapeutic efficacy is crucial, with advances in non-viral delivery methods and novel vector designs showing promise (Wang et al., 2020). Combining gene therapy with pharmacological agents or stem cell therapy may enhance therapeutic outcomes and provide comprehensive treatment strategies for DR (Whitehead et al., 2018). Advances in genomic technologies and precision medicine will enable personalized gene therapies tailored to individual genetic profiles, improving treatment efficacy and reducing adverse effects (Yue et al., 2019).

Conducting long-term studies to assess the durability and stability of gene therapy effects is essential, as understanding long-term implications and potential need for repeat treatments will guide clinical practice (Campa et al., 2017). Establishing robust ethical and regulatory frameworks to oversee gene therapy development and implementation is critical, ensuring patient safety and addressing ethical concerns to facilitate adoption (Bulaklak and Gersbach, 2020). Addressing cost and accessibility issues associated with gene therapy is vital to ensure treatments are available worldwide, regardless of socioeconomic status (Tan et al., 2021).

By overcoming these challenges and advancing our understanding of DR’s genetic and molecular underpinnings, gene therapy has the potential to revolutionize DR management, offering new hope for patients suffering from this debilitating disease (Ku and Pennesi, 2015).

### 4.3 Individualized management using genetic information

#### 4.3.1 Integration of genetic insights in clinical management

**FIGURE 4:**
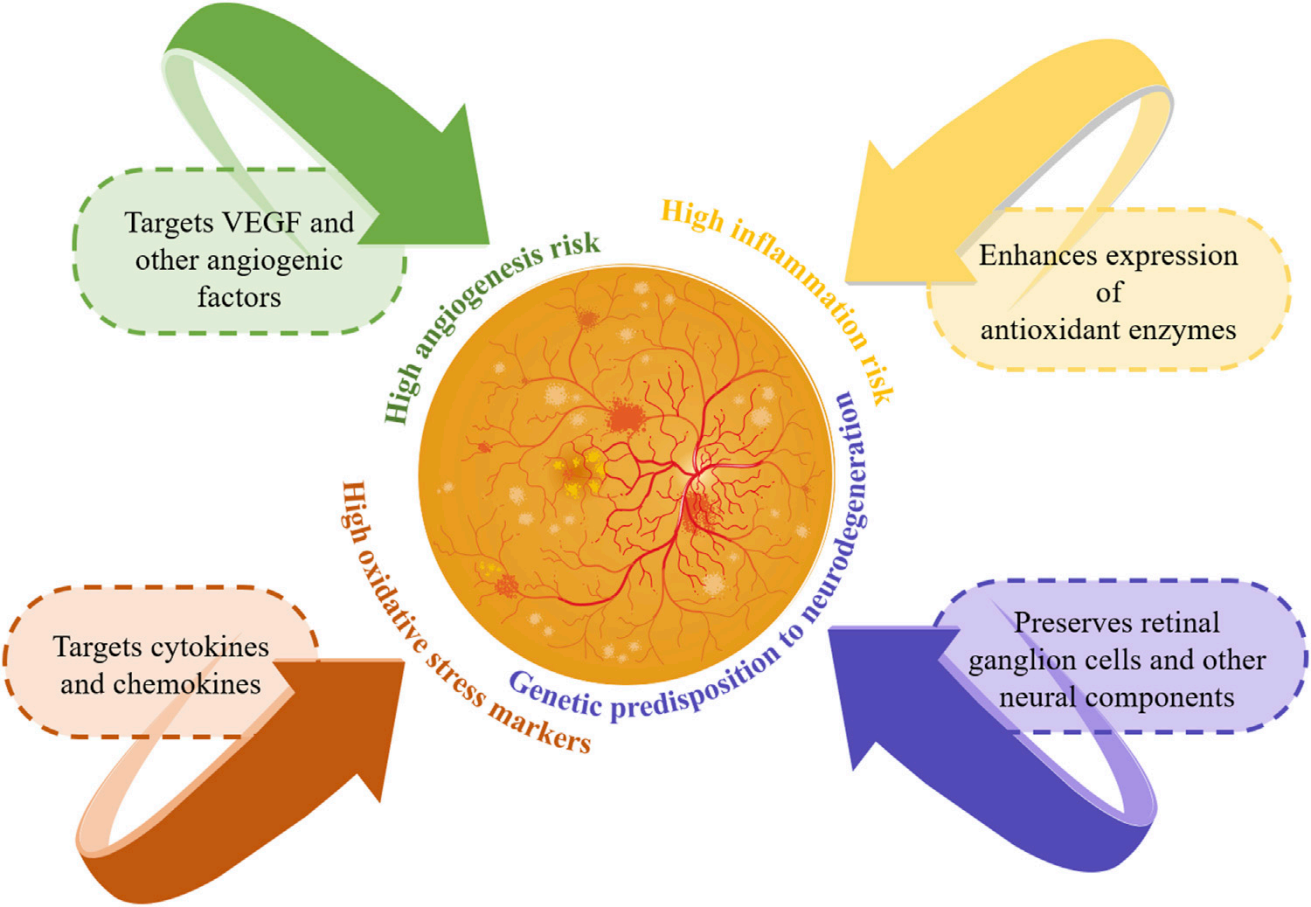
Personalized management strategies based on genetic information. This figure illustrates the personalized management of DR using genetic information. It highlights genetic risks and targeted treatment strategies: targeting VEGF for high angiogenesis risk, cytokines for high inflammation risk, enhancing antioxidants for high oxidative stress, and preserving retinal cells for neurodegeneration risk. These personalized strategies improve early detection and intervention, enhancing patient outcomes in DR.

Integrating genetic information into DR management heralds a transformative era of personalized medicine, offering new avenues for patients with this complication. As the principal cause of vision loss among working-age adults, DR’s complex genetic interactions are unveiled through genomics, setting the stage for customized treatment solutions. Research into genetic predisposition has identified genes linked to DR risk, progression, and severity, including those involved in angiogenesis (VEGFA), inflammation (IL-6, TNF-α), oxidative stress (SOD1, SOD2), and glucose metabolism (AKR1B1). This genetic insight facilitates early detection of at-risk patients or those progressing to severe stages like proliferative DR or diabetic macular edema, enabling prompt intervention (Hampton et al., 2015). Figure 4 illustrates personalized treatment strategies based on genetic information.

Utilizing genetic insights for DR clinical management enables a nuanced treatment approach. For example, preemptively applying anti-VEGF therapy in individuals genetically inclined to high angiogenesis or using anti-inflammatory drugs for those susceptible to inflammation can significantly improve patient outcomes (Simó and Hernández, 2015). Gene therapy is emerging as a pioneering field, targeting curative outcomes by altering the genetic framework underlying DR. Despite this optimism, challenges such as comprehensive genetic screening, ethical dilemmas, and the socio-economic impact of genetic data on insurance and employment persist and require further scrutiny (Mishra et al., 2016).

The journey of personalized DR treatment stands to benefit from genomic advances that will reveal additional genetic markers tied to disease risk and treatment efficacy. Bioinformatics and machine learning play pivotal roles in decoding genetic intricacies, refining the feasibility of tailored therapeutic strategies (Cabrera et al., 2020b). Emerging technologies like CRISPR-Cas9 for precise genome editing hold the promise of correcting genetic defects at their source, potentially providing long-term solutions for managing DR (Wang et al., 2017). Advances in NGS enhance our ability to identify rare genetic variants that contribute to DR susceptibility and progression (Shtir et al., 2016). These tools improve our understanding of the genetic basis of DR and pave the way for new therapeutic targets and personalized interventions.

#### 4.3.2 Future prospects and emerging technologies

The future of individualized management using genetic information in DR looks promising, with emerging technologies and research directions set to revolutionize the field. CRISPR-Cas9 technology offers the potential to directly correct genetic mutations associated with DR. This precise genome editing tool can target specific genes involved in DR pathogenesis, providing a long-term solution beyond symptom management (Whitehead et al., 2018). Integrating advanced bioinformatics and machine learning algorithms is crucial for analyzing large-scale genetic data. These technologies can identify complex patterns and interactions among multiple genetic variants, leading to more accurate risk prediction models and personalized treatment plans.

Advances in NGS technology enable the identification of rare genetic variants that may contribute to DR. This comprehensive genetic profiling can uncover novel therapeutic targets and enhance our understanding of the genetic architecture of DR (Cheung et al., 2016). Research into epigenetic modifications and their role in DR is expanding. Epigenetic therapies targeting these modifications offer a promising new avenue for treatment by reversing aberrant gene expression patterns associated with DR (Santovito et al., 2021). Personalized medicine extends to pharmacogenomics, where genetic information predicts individual responses to specific drugs. This approach can optimize treatment efficacy and minimize adverse effects by tailoring drug selection and dosing to the patient’s genetic profile (Agarwal et al., 2016).

The ultimate goal is to integrate genetic testing and personalized management strategies into routine clinical practice. This requires developing standardized protocols and guidelines to ensure the effective and ethical use of genetic information in patient care. Overcoming these challenges and advancing our understanding of DR’s genetic and molecular underpinnings, gene therapy and personalized medicine could revolutionize the management of diabetic retinopathy, offering new hope for patients suffering from this debilitating disease. The convergence of cutting-edge genetic research, innovative technologies, and personalized therapeutic approaches indicates a promising future for the individualized management of DR.

## 5 Challenges and opportunities: translating genetic findings into diabetic retinopathy therapeutic strategies

Translating genetic discoveries into practical DR treatment strategies presents significant challenges and opportunities. Gene therapy promises to revolutionize treatment paradigms by potentially providing durable therapeutic effects with fewer administrations. However, transitioning these therapies from the laboratory to the clinic is hampered by significant efficacy and safety concerns. This underscores the urgent need to refine delivery vectors and establish comprehensive regulatory frameworks to ensure safe clinical application (Wang et al., 2020).

Stem cell therapy research also illustrates the complexity of translating basic science into clinical practice. Issues like stem cell heterogeneity and challenges in effective cell homing suggest that, despite its potential, the field requires more robust clinical trials to verify therapeutic viability and safety (Li et al., 2021). Similarly, integrating nanotechnology into DR treatments faces hurdles. Current nanotechnology-based drug delivery systems have shown limited success in human trials, despite promising results in animal studies, highlighting a critical gap in translating laboratory success into clinical utility (Liu and Wu, 2021).

Additionally, epigenetic modifications offer a new therapeutic frontier by potentially reversing deleterious epigenetic changes. However, the success of these strategies depends on early DR diagnosis and the application of treatments that can effectively modify epigenetic patterns (Kumari et al., 2020).

To overcome these barriers, the scientific community must: 1) Develop and validate advanced gene delivery systems that can safely and efficiently target retinal cells (Wang et al., 2020). 2) Conduct extensive clinical trials to determine the efficacy and safety of stem cell- and nanotechnology-based therapies (Li et al., 2021; Liu and Wu, 2021). 3) Improve diagnostic capabilities to detect DR at earlier stages and thus improve the efficacy of epigenetic treatments (Kumari et al., 2020).

These efforts should be complemented by strong interdisciplinary collaborations that integrate insights from genetics, molecular biology, pharmacology, and clinical sciences to streamline the translation of research findings into effective DR therapies. These strategies promise to bridge the gap between experimental research and clinical application, paving the way for more personalized and effective DR management solutions.

## 6 Future directions for research

### 6.1 Expanding genetic studies and profiling

In DR research, significant progress has been made in genetic studies, yet translating these discoveries into clinical applications presents multifaceted challenges. Future research must clarify the specific contributions of genetic variations to DR risk and explore the interactions between genetic and environmental factors. Developing precise genetic risk assessment tools and clinically validating personalized treatment strategies are crucial. Addressing ethical, legal, and social issues is paramount for advancing the field (Wang et al., 2020).

Comprehensive genetic profiling and risk prediction remain incomplete, with several genetic loci associated with DR identified, but the full genetic landscape yet to be clarified. Future studies should focus on large-scale GWAS and whole-genome sequencing to identify more risk alleles, especially those contributing to individual susceptibility and disease variability. Identifying genetic variations associated with DR marks the beginning of a journey. Future research should delve into functional genomics to understand how these variations affect gene expression and lead to DR pathophysiology (Skol et al., 2020). Studying the molecular pathways and networks affected by these genes, such as angiogenesis, inflammation, and oxidative stress, will be vital.

### 6.2 Integrating pharmacogenomics and personalized medicine

The variability in responses to current DR treatments, such as anti-VEGF injections, underscores the need for personalized medicine (Agarwal et al., 2016). Future research should explore pharmacogenomics to identify genetic markers predictive of treatment responses or adverse reactions. Advancements in gene therapy and CRISPR-Cas9 gene editing technologies could lead to revolutionary treatment methods for DR (Wang et al., 2017). Targeting specific genetic mutations or maladaptive gene expression patterns responsible for DR could provide long-term solutions for preventing or reversing the disease (Rasoulinejad and Maroufi, 2021).

### 6.3 Considering systemic factors and ethical implications

Given the systemic nature of diabetes and its complications, genetic research into DR should not be isolated. Studies should also consider the genetic aspects of diabetes management, as these are directly related to DR progression (Sivaprasad et al., 2023). Understanding the genetic factors that influence both diabetes and its ocular complications will provide a more holistic approach to treatment and prevention.

As genetic research in DR advances, considering the ethical, legal, and social implications of genetic testing and personalized interventions is essential. Future research should address potential genetic discrimination, privacy concerns, and equitable access to genetic testing and treatments. Ensuring that advancements benefit all patients, regardless of socioeconomic status, is crucial for the ethical application of genetic discoveries in DR. Collaborative efforts among researchers, clinicians, ethicists, and policymakers will be vital to navigate these challenges effectively.

### 6.4 Advancing diagnostic and therapeutic approaches

To overcome existing barriers, the scientific community must focus on several key areas: developing and validating advanced gene delivery systems that can safely and efficiently target retinal cells; conducting extensive clinical trials to determine the efficacy and safety of stem cell- and nanotechnology-based therapies; and enhancing diagnostic capabilities to detect DR at earlier stages, which can significantly improve the efficacy of epigenetic and other emerging treatments (Yun et al., 2020). Advanced imaging technologies and biomarkers are vital for early diagnosis and monitoring treatment response (Shao et al., 2020).

## 7 Conclusion

Genetics is fundamental to understanding and managing DR, revealing molecular mechanisms and enabling personalized medicine. Identifying genetic variations has advanced our knowledge of disease progression, enabled early diagnosis, and facilitated tailored treatments, thus improving patient outcomes and reducing side effects. This review highlights key genetic markers associated with DR, enhancing risk assessment and early intervention, and demonstrates that applying these insights in clinical practice can revolutionize DR management with targeted therapies.

Challenges remain, including uncovering unknown genetic variations, understanding gene-environment interactions, and addressing ethical concerns regarding genetic information. Future research promises further advancements in genetic markers and therapeutic targets, refining diagnostic and therapeutic techniques. Interdisciplinary collaborations are essential to translating genetic discoveries into clinical practice. Researchers, clinicians, and bioinformaticians must work together to validate findings, develop applications, and address ethical issues.

In conclusion, genetics plays a critical role in DR, and future innovations will continue to enhance diagnostic and therapeutic techniques. Ongoing research and collaboration are vital for enhancing patient outcomes globally. Clinicians should integrate genetic testing into DR screening and consider genetic profiles in treatment plans. Researchers should focus on identifying novel genetic markers and exploring gene-environment interactions.
